# A Rare Case of Paraesophageal Hernia Repair Complicated by Pancreatic Injury

**DOI:** 10.7759/cureus.37381

**Published:** 2023-04-10

**Authors:** Bianca Varda, Jake Jasurda, Abdul Haseeb

**Affiliations:** 1 Internal Medicine, Loyola University Medical Center, Maywood, USA; 2 Gastroenterology and Hepatology, Loyola University Medical Center, Maywood, USA; 3 Gastroenterology, Loyola University Medical Center, Maywood, USA

**Keywords:** gastroenterology and endoscopy, gastroesophageal reflux disease (gerd), endoscopic ultrasound (eus), esophagogastroduodenoscopy (egd), endoscopic retrograde cholangiopancreatography (ercp), pancreas endosopy, pancreatic leak, laparoscopic nissen’s fundoplication, paraesophageal hiatal hernia, hiatal hernia

## Abstract

Esophageal hernias are anatomical defect that affects up to 50% of the population. While they may be asymptomatic, hernias may also result in reflux and dysphagia, among other symptoms. In such cases, hernia repair is warranted. The most common type of repair is laparoscopic Nissen fundoplication, which is usually well-tolerated. Herein, we present a rare case of paraesophageal hernia repair complicated by pancreatic injury and pancreatic leak.

## Introduction

Hiatal hernias are a fairly common phenomenon. There are four types of esophageal hernias, and each may present with a variety of symptoms ranging from reflux to dysphagia. Complications include gastric volvulus, gastritis, and respiratory complications due to lung compression if the hernia is large. A diagnosis is made via barium swallow study or endoscopy. Management consists of observation for sliding hiatal hernias. For paraesophageal hernias, surgery is considered based on the type and size of the hernia and the presence of symptoms. Pancreatic involvement seen in association with hiatal hernias is rare. A PubMed search using the keywords “hiatal hernia + pancreas” yielded only 15 results. Here, we present a case of a rare complication of hiatal hernia repair resulting in pancreatic injury and leak. This case was previously presented at the 2022 ACG Meeting on October 25, 2022.

## Case presentation

**Figure 1:**
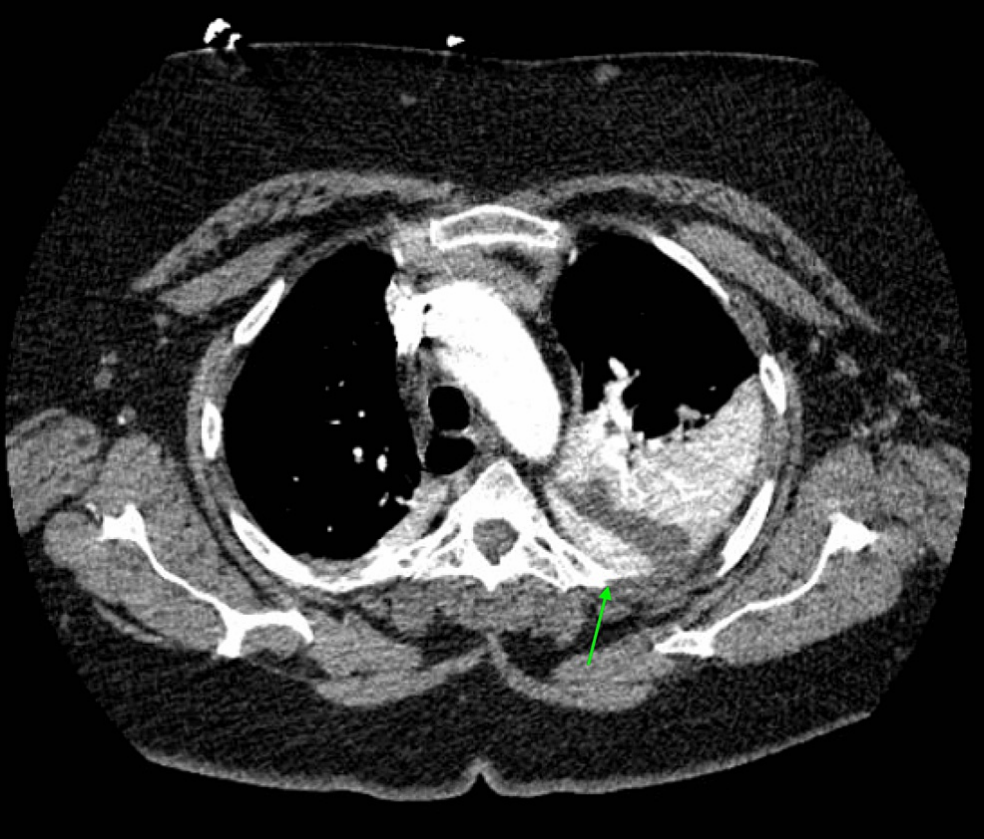
CT PE showing a large left pleural effusion extending across the midline with mass effect causing multifocal atelectasis and trace pneumomediastinum

A 58-year-old female with hypertension, gastroesophageal reflux disease (GERD), and a type III paraesophageal hernia presented to the General Surgery clinic due to months of decreased oral intake secondary to worsening dysphagia, belching, and gas. She subsequently underwent laparoscopic Nissen fundoplication with gastroscopy. On the day of the operation, she was afebrile, and her vital signs were stable. The physical examination was unremarkable, and the surgery went well. However, two days postoperatively, she developed a worsening respiratory status, requiring intubation for three days. Computed tomography (CT) pulmonary embolism (PE) showed no PE, a large left pleural effusion extending across the midline of the abdomen with mass effect causing multifocal atelectasis, and trace pneumomediastinum (Figure 1). Overall, these findings were concerning for an esophageal anastomotic leak. The barium swallow study performed the following day was normal, with no evidence of anastomotic leak or esophageal perforation.

**Figure 2:**
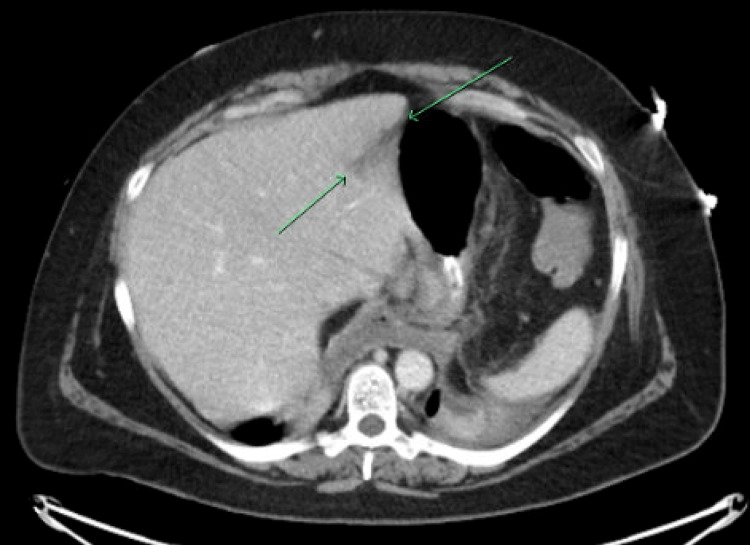
CT C/A/P demonstrating peripancreatic stranding and fluid with a hypodense band within the pancreas, concerning for focal injury and pancreatic leak.

Unfortunately, the patient’s clinical course continued to deteriorate. Her course was complicated by acute hypoxic respiratory failure requiring intubation on postoperative day three. A left pigtail catheter was placed for the pleural effusion, resulting in an improvement in that fluid collection. She began to improve and was successfully extubated over the next few days. However, on post-op day nine, her white blood cell count increased to 19.6 K/uL, and she had intermittent fevers with a maximum temperature of 38.8C. A repeat CT chest/abdomen/pelvis (C/A/P) showed peripancreatic stranding and fluid with a hypodense band within the pancreas, concerning focal injury and pancreatic leak (Figure 2). At that time, gastroenterology was consulted for further evaluation of the pancreatic injury. The patient underwent endoscopic retrograde cholangiopancreatography (ERCP) on post-op day 10. Although no contrast extravasation was visualized, due to high clinical suspicion of the pancreatic leak, a sphincterotomy was performed, and a pancreatic duct stent was placed. She was treated with empiric antibiotics for sepsis, although cultures remained negative, and no source of infection was found. She continued to improve with the resolution of her respiratory distress.

**Figure 3:**
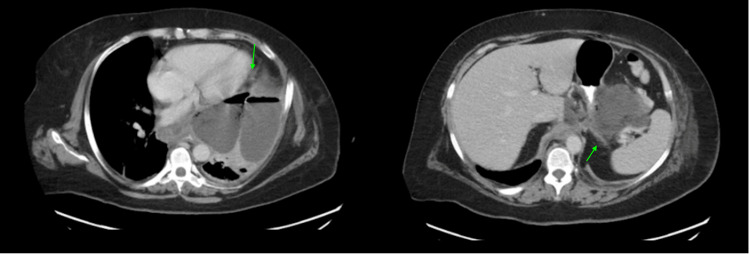
Repeat CT C/A/P revealed a mild interval increase in the peripancreatic fluid collection size concerning for persistent leak, increasing mass effect on cardiomediastinal structures, and loculated thoracoabdominal fluid collections.

One week later, she returned to the clinic due to fevers, worsening dyspnea, and continued poor oral intake and was directly admitted to the hospital. On admission, she required supplemental oxygen via nasal cannula but was otherwise hemodynamically stable. Repeat CT C/A/P revealed a mild interval increase in the peripancreatic fluid collection size concerning for persistent leak, increasing mass effect on cardiomediastinal structures, and loculated thoracoabdominal fluid collections (Figure 3). For further management of the fluid collections, the patient underwent drain placement of the pleural fluid and left lower quadrant fluid collections with Interventional Radiology. Fluid cultures grew Enterobacter cloacae, Prevotella melaninogenica, and Strep viridins, and she was treated with a three-week course of cefepime and metronidazole. Although the fluid collections initially improved, they recurred after drain removal, and she required repeat drain placement. She also underwent EGD with endoscopic ultrasound (EGD with EUS) for necrosectomy and cyst gastrostomy tube placement. Her hospital course was complicated by complex empyema, and she ultimately required decortication with Thoracic Surgery. She improved following the operation and was discharged on IV antibiotics for further treatment.

## Discussion

Hiatal hernias affect anywhere from 10% to 50% of the population [1]. Hiatal hernias (HH) occur due to a defect in the development of the diaphragm. Type I, or sliding, hiatal hernias are the most common. They occur due to weakened muscles and ligaments at the gastric cardia, causing the cardia to move upwards into the posterior mediastinum [1]. Type I hiatal hernias most often present with symptoms of gastroesophageal reflux disease (GERD).

Type II hiatal hernias, also referred to as paraesophageal, occur due to the failure of the peritoneal sac in the esophageal hiatus to regress during development. Instead, it bulges into the posterior mediastinum. This type of hernia most commonly occurs in older adults. However, because the gastroesophageal junction (GEJ) remains in place in this type of hernia, symptoms of reflux are much rarer, but patients may present with early satiety, dyspnea, or postprandial pain [1].

Type III hiatal hernias are termed mixed, as they are both sliding and paraesophageal hiatal hernias and, therefore, present with mixed symptoms. Type IV hiatal hernias are not clearly defined but are referred to as giant paraesophageal hernias and are diagnosed when more than 33.33% to 50% of the stomach herniates through the esophageal hiatus [1]. Common symptoms of type IV hiatal hernias include postprandial pain, dysphagia, and iron deficiency anemia (IDA). Given the extent of stomach or abdominal viscera that may herniate into the thoracic cavity, patients may also experience shortness of breath [1,2].

Diagnosis of hiatal hernias usually occurs incidentally when symptoms prompt further imaging studies, such as chest radiography, which may show a coiled nasogastric tube or a low retrocardiac air-fluid level [2]. A barium swallow is another form of diagnosis. The criteria for diagnosing a sliding hiatal hernia on barium swallow is a greater than 2cm separation between the diaphragmatic hiatus and stomach rugae. Finally, endoscopy may reveal a hiatal hernia [2].

Due to the association between hiatal hernias and GERD, patients are also at risk for esophagitis, Barrett’s esophagus (BE), and esophageal adenocarcinoma (EAC) [3]. Complications from unrepaired hernias include intestinal obstruction and strangulation, both of which can be very severe. Gastric necrosis and perforation are the main causes of mortality secondary to HH [4]. Based on the type and size of the hernia in combination with the patient’s symptoms, hernia repair may be indicated. If patients are symptomatic with a paraesophageal hernia and are deemed fit for surgery, laparoscopic HH repair is the standard [5].

One study by Chang and Thackeray (2016) reviewed laparoscopic HH repair in 221 patients over a five-year period. Overall, most surgical complications were minor, such as nausea. One patient suffered a hemorrhage one-week postoperatively. The study concluded that laparoscopic repair with biologic mesh was efficacious and safe, with or without simultaneous bariatric and/or antireflux procedures [6]. During the procedure, the hernia sac is reduced and excised, and the esophagus is mobilized before the esophageal hiatus is closed [2]. Robotic HH repair has also become common. Outcomes seem to be comparable to laparoscopic repair, with some studies demonstrating a shorter length of hospitalization after robotic repair [7].

Complications of HH repair include esophageal and gastric perforation and pneumothorax, among others [8]. Furthermore, paraesophageal hiatal hernias have a high rate of recurrence [8]. While these complications may occur, overall laparoscopic repair remains the treatment method of choice and is generally well tolerated [9]. There are very few reports of pancreatic involvement in cases of hiatal hernias. Furthermore, a majority of these reports describe herniation of the pancreas through the HH [10,11]. 

## Conclusions

To the best of our knowledge, this is one of the only reports of paraesophageal hernia repair complicated by a pancreatic leak. This case illustrates a very rare post-HH repair clinical course. While our patient’s initial clinical symptoms were very typical for a large paraesophageal hernia, her postoperative course was complicated by pancreatic injury and a subsequent leak, with eventual sepsis resulting in a prolonged recovery. It is important to keep such cases in mind and be diligent regarding post-operative follow-up to identify any possible complications.
